# Change and Continuity in Preventive Practices Across the COVID-19 Pandemic Among Rural and Urban Latinx Immigrant Worker Families

**DOI:** 10.3390/hygiene2040018

**Published:** 2022-11-10

**Authors:** Sara A. Quandt, Sydney A. Smith, Jennifer W. Talton, Haiying Chen, Paul J. Laurienti, Thomas A. Arcury

**Affiliations:** 1 Department of Epidemiology and Prevention, Division of Public Health Sciences, Wake Forest University School of Medicine, Winston-Salem, NC 27157, USA; 2 Department of Biostatistics and Data Science, Division of Public Health Sciences, Wake Forest University School of Medicine, Winston-Salem, NC 27157, USA; 3 Department of Radiology, Wake Forest University School of Medicine, Winston-Salem, NC 27157, USA; 4 Department of Family and Community Medicine, Wake Forest University School of Medicine, Winston-Salem, NC 27157, USA

**Keywords:** coronavirus, prevention, essential workers, farmworkers, Latino, repeated measures

## Abstract

**Background:**

The COVID-19 pandemic has put essential workers at high risk for contracting the disease. This study documents situational compliance with public health recommendations such as masking and social distancing among rural and urban Latinx families, with the goal of understanding change over time in COVID-19 risk reduction behaviors.

**Methods:**

Respondents for 67 rural families and 44 urban families responded to repeated telephone surveys at three time points in the first year of the pandemic, providing data on use of masks and social distancing by themselves and family members while interacting with others at home, work, and in the community. Cumulative logistic regression models were employed to compare changes in risk behaviors between rural and urban groups over time.

**Results:**

While group descriptive results indicated behaviors that posed low risk at each time point, regression models revealed greater change between time points for rural than urban residents. Rural residents also had gendered patterns.

**Conclusions:**

Patterns of change appear to reflect structural issues such as seasonal labor demand and gender roles more than pandemic fatigue or changing public health recommendations. The findings suggest that structural factors play a role in individuals complying with public health prevention measures for COVID-19.

## 1. Introduction

Essential workers across the United States have experienced high rates of morbidity and mortality during the COVID-19 pandemic [1-2]. These include workers in both urban and rural communities employed in agriculture, food production, construction, and service industries [3-5]. Their occupations do not allow work-from-home and often do not require the type of protective measures advocated to reduce the spread of the virus. In addition, lack of paid sick leave and protections for missed work, as well as being required to work despite illness and living in crowded housing, have prevented some workers from quarantining when exposed or isolating when infected [4, 6-8]. Because many essential jobs are held by racial and ethnic minority workers, mortality and morbidity both directly and indirectly related to COVID-19 have disproportionately affected these population segments [3, 9-12] and are likely reflected in national statistics for COVID-19 by race and ethnicity. For example, Hispanic or Latino persons were 1.5 times as likely to be a COVID-19 case, 2.2 times as likely to be hospitalized, and 1.8 times as likely to die, compared to non-Hispanic whites [13].

Public health recommendations to reduce COVID-19 transmission, though initially varied [14], settled on masking and social distancing by May of 2020 [15-17]. The practice of mask-wearing was initially advocated to reduce the transmission of COVID-19 to the mask wearer from others, but subsequent research demonstrated that masking also protected other persons from exposure to the virus spread by the mask wearer [18-19]. Social distancing was advocated to reduce transmission of the virus once it was established that the virus could remain airborne in aerosolized droplets. Although conflicting recommendations were put forth in the US, national recommendations solidified by May 2020, that distances between people of six feet should be maintained both indoors and outdoors to prevent exposure [20]. Some mandates were issued, particularly for masking in public settings, but much prevention was an individual decision. Data collected early in the pandemic showed that, while knowledge of COVID-19 etiology and prevention was high, preventive behaviors were not consistently practiced [21]. Research across multiple countries showed that factors behind practicing prevention were related to demographic factors (sex, age), beliefs (liberal political beliefs, more empathy) and self-perceptions about health [17, 22-23].

With the ongoing and uncertain nature of the pandemic, healthcare and governmental personnel feared that “caution fatigue” or “pandemic fatigue” [24] would reduce compliance with protective measures. Attempts to look for such behavioral trends have used mixed approaches (e.g., repeated measures panel designs [17, 23, 25], repeated cross-sectional designs [22, 26-27]) and have generally used broad measures of compliance with COVID-19-related protective behaviors (e.g., “Are you following the recommendations of authorities to prevent spread of COVID-19?” [25]). Almost all are focused on psychological factors that could affect compliance, none target marginalized populations. Not surprisingly, especially because studies include different countries, results have been mixed and hard to generalize across studies.

The present study uses longitudinal data gathered in three repeated surveys from a marginalized Latinx population composed largely of essential workers and their families. It focused on situational compliance (at home, in the homes of others, at work, and at large social events) with two widely recommended practices (mask wearing and maintaining social distance). Using these data, we examine continuity and change during the first year of the pandemic in the practice of public health measures advocated to reduce exposure to COVID-19. Parents in Latinx households in North Carolina report for themselves, their children, and their spouse/partner, at home, in the homes of others, at work, and at special events. Data were gathered in Spring 2020, Fall 2020, and Winter 2021, providing an opportunity to understand change over time as public health messaging intensified and the morbidity and mortality rates from COVID increased. These data help shed light on the uptake of public health behaviors during a pandemic.

The paper’s specific aims are: (1) to describe COVID-19 prevention practices at three points in time for urban and rural Latinx households; and (2) to model these practices and examine the role of time and urban/rural residence in their continuity and change.

## 2. Materials and Methods

The study reported here is part of a larger two-group, prospective study examining the health and cognitive effects of pesticide exposure in children. The larger study uses a comparative design, with a sample of families of rural Latinx farmworkers with children and a sample of similar urban families, but without any farmworker members. Additional details of the study can be found elsewhere [21, 28]. The current study used a telephone survey to reach a parent of the children in these families three times after the start of the COVID-19 pandemic: in Spring, 2020; Fall, 2020; and Winter, 2021. All procedures for both the original study and this COVID-19 study were approved by the Wake Forest University Institutional Review Board. All families completed informed consent/assent to participate in the main study and verbal consent to participate in the COVID-19 telephone questionnaire survey. The study received a Certificate of Confidentiality from the National Institutes of Health.

### 2.1. Inclusion Criteria and Participant Recruitment

Inclusion criteria for the families were similar in both samples when recruited from March, 2018, to December, 2019; they reflect the purpose of the larger study. Each family had to have a child aged 8 years at baseline who had completed the first grade in the US. All children had to be from families that self-identified as Latino or Hispanic, and with household incomes below 200% of the US federal poverty guideline. In the rural sample, the mother or her partner must have been employed in farm work on non-organic farms during the past three years. In the urban sample, no adults could have been employed in any industry that involves routine exposure to pesticides (e.g., farm work, landscaping, pest control) in the previous three years. Families in the urban sample could not have lived adjacent to agricultural fields in the previous three years.

Exclusion criteria for both samples included children having life-threatening illnesses, prior history of neurological conditions, physical condition or development disorder that would not allow them to complete or would interfere with the results of neurobehavioral tests or MRIs (used in the larger main study), primary language other than Spanish or English spoken in the home, or refusal of mother/guardian to complete the questionnaires.

In the larger study, a total of 76 children were recruited for the rural sample and 65 children for the urban sample. For the recruitment of the rural children, the community partner North Carolina Farmworkers Project developed a list of farmworker families with an 8 year old child, and the locations where they lived. In addition, other community organizations that served farmworker families in the recruitment area were contacted. Study personnel contacted the parents or guardians. Similarly, for the original urban sample, local recruiters in Winston-Salem, NC and surrounding areas and community members developed a list. For both samples, parents or guardians were contacted by a bilingual staff member who explained the overall study procedures, answered questions, and, if they agreed to participate, obtained signed informed consent from a parent/guardian and assent from the child.

Prior to the first COVID-19 telephone survey, 5 children in the rural sample and 17 in the urban sample withdrew, moved away from the study area, or were lost to follow-up. The remaining children represented 67 rural families and 44 urban families, because some families had more than one child enrolled. For the first telephone sample, 2 families refused to participate and 5 could not be reached, all in the urban sample. A total of 67 rural families and 37 urban families could be reached and agreed to participate. In the second and third telephone survey, 63 and 40 rural families and 64 and 41 urban families, respectively, participated. In all, 63 rural and 34 urban families (total=97) participated in all three data collection surveys. Additional families participated in 1 (n=6) or 2 (n=8) of the surveys.

### 2.2. Data Collection

Data for this study were gathered using a telephone survey during these dates: May 1 to June 5, 2020, with 98.1% collected in May; September 22 to December 16, 2020, with 82.7% collected in September and October; and January 9 to March 12, 2021, with 98.1% collected in January and February. In this paper, the surveys are labeled Spring 2020, Fall 2020, and Winter 2021. Recruitment and interview procedures have been described elsewhere [21, 27]. Mothers were the participating parent in all but two of the surveys. If the mother agreed to participate, her informed consent was noted, and the interviewer proceeded to conduct a standardized interviewer-administered questionnaire in the language of the participant’s choice (English or Spanish) using a tablet. Two rural fathers each completed a survey for mothers who were unavailable in the Fall 2020 survey. Informed consent was obtained from both. Data from these two surveys were recoded as described below, and this paper refers to “mothers” as these fathers answered in place of the mothers. Data were entered in real time during the interviews using Research Electronic Data Capture (REDCap). REDCap is hosted at Wake Forest School of Medicine through the Clinical and Translational Science Institute. The REDCap system provides secure, web-based applications for various types of research [29]. Data from these interviews were later merged with selected personal, family, and household variables collected in the main study questionnaires.

Questionnaire items relating to the coronavirus and COVID-19 were adapted from existing studies (e.g., [30]), where available, or from questions recommended for COVID-19 research by governmental and non-governmental agencies. Because of the need for rapid data collection, validation was limited to checks on face validity and interviewer reports of difficulties experienced by respondents during practice interviews.

### 2.3 Variables and Measures

Variables from the main study baseline questionnaire were used to create measures to describe the sample. These included the following measures for the mother: age (age at first COVID-19 survey, calculated from baseline data) (25-29, 30-34, 35-39, and 40-47 years), country of origin (Mexico, El Salvador, Guatemala, Honduras, Venezuela, and US), educational attainment for self and spouse/partner (<grade 6, grades 6-8, grades 9-11, high school or higher), preferred language for conversation (Spanish, English, an indigenous language), household size, and current occupation. Group assignment of the family to the rural or urban work sample was also noted from the baseline questionnaire.

Data were collected about physical distancing and mask wearing during the past week at each of the three time points. These data were used to construct nine outcome measures of COVID-19 risk. In the two instances where fathers reported, their responses were recoded so that their responses for themselves became the responses for spouse/partner, and their responses for spouse/partner became responses for the mother (also referred to hereafter as the respondent).

Respondents were asked how many adults had visited in the respondent’s house in the past week. Response options were none, 1 or 2, 3 or 4, and 5 or more. This question was also asked about child visitors. Respondents were also asked how many different houses, apartments, or trailers of others they had visited in the last week. Response options were none, 1 or 2, 3 or 4, and 5 or more. Similar questions were asked about the household children and the respondent’s spouse/partner. Respondents were asked about mask wearing during visiting, with response options being that masks were worn by all, some, or no persons present during visits.

Respondents were asked how many people they worked with, defined as the number of persons with whom they worked closely enough to have a normal conversation for at least some of the work time. Response options were none, 1 or 2, 3 or 4, and 5 or more. Mask use was queried for co-workers, with response options of all of them, some of them, and none of them wore masks at work. Similar questions were asked for the spouse/partner at work.

To obtain information on large social gatherings in the past week, respondents were asked if any household member had attended church and the approximate number of attendees. The same set of questions was asked about whether any household member had attended a party or other social event such as a cookout, baptism, quinceañera, wedding, or funeral in the past week. For both large social gatherings, mask use of participants was queried with response options of all of them, some of them, and none of the attendees wore masks.

Risk measures were calculated for visit (5 risk measures), work queries (2 risk measures), and large social gatherings (2 risk measures). In cases where there were no visits with others, no close workplace interactions, or no church/social event attendance, risk was classified as low risk. Where any visiting, workplace interaction, or church/social events occurred, risk was classified as medium (masks worn some or all of the time) or high (masks worn none of the time).

For the seven visit and large social gathering measures, there were few records in the high or medium risk category for one or both of the rural and urban groups. We combined the medium and high risk categories for these seven measures and kept the risk at three levels for respondent and spouse workplace. For the seven measures, risk became defined as low (no visits or social gatherings) or medium/high (any visit or social gathering). The mask responses became inconsequential because any respondents who reported visits or attending events would be in the medium/high risk group regardless of how often masks were worn. Because low risk was defined as no visits or social outings, we reclassified this as no risk and the alternative as any risk.

For the work questions, risk was kept classified as no/low (does not work or works with 0 persons close enough for normal conversation), medium (works with others close enough for normal conversation and masks are worn some or all of the time), or high (works with others close enough for normal conversation and masks are worn none of the time). Seven participants (1 rural and 5 urban in Spring 2020, and 1 urban in Winter 2021) did not know about mask wearing for spouse’s workplace. These responses were classified as medium risk.

### 2.4. Data Analysis

Descriptive statistics (counts, percentages) were calculated by rural status for mothers’ individual and household characteristics as well as categorized risk behavior across three COVID-19 questionnaires. Tests between groups were performed with Chi-square or Fishers Exact test, as appropriate. The outcomes were categorized as three levels of risk (low, medium, and high) or collapsed into two levels of risk (none and any) when few or no responses were reported in the medium or high risk categories. We employed cumulative logistic regression models to compare the changes in risk behaviors over time between the groups. The models included the interactive effect of time and group to allow comparisons between groups at each time point, as well as comparisons across time points within each group. However, no urban families reported any event attendance in Winter 2021; and the cumulative logistic regression model could not estimate odds ratio with the interactive effect of time and group. When sparse risk counts and the interaction term affected the model fit, only main effects of time and group were included in the model to carry out overall comparisons between group and across time points. P-values of less than 0.05 were considered statistically significant.

## 3. Results

### 3.1. Description of the Sample

Respondents were women and ranged in age from 25 to 47 years (Table 1). Over 90% of both samples were born in Latin America, with Mexico being the most common country. Spanish was the preferred language for most. Years of formal education for the respondents ranged from 0 to college graduate, with the median in both samples being ninth grade. The spouse/partners for both samples had slightly less education than the respondents. The two samples differed in country of origin (p=0.0146) and education of spouse/partners (0.0349).

At the first COVID-19 questionnaire available, total household sizes were generally large, ranging from 1 to 10 (median=5) and 3 to 13 (median=6) in the rural and urban samples, respectively. For the rural sample, the number of adults in the household ranged from 1 to 6, while the number of children ranged from 0 (a respondent currently separated from her family) to 7. For the urban sample, the ranges were 1 to 4 for adults and 1 to 10 for children.

At study baseline, rural families reported that the most common industry in which women worked was agriculture (45%), followed by not currently working (18%); for men, it was construction (39%), followed by agriculture (32%). For urban families, most women were not in the labor force (41%), and the majority of men worked in construction (68%).

### 3.2. Description of Risk Behaviors at Each Time Point

For measures of hosting visitors in homes or visiting in other homes, the rural sample appeared to follow predominantly practices associated with no risk of COVID-19, with the proportion at any risk declining over time (Table 2). The urban sample also reported predominantly no risk behaviors, but without consistent change over time. Measures of risk at work for oneself fell largely in the low risk category, particularly for urban women. The predominance of low risk likely reflects the large number of urban women who do not report work outside the home. The risk at work for the spouse was higher with measures that fell largely in the medium risk category. Respondents reported relatively little attendance at church and other group events over the pandemic.

### 3.3. Comparison of Risk Measures over Three Time Points

When the 5 outcomes related to visiting across households as well as the church attendance measure were modeled, the rural by time interactions were not significant. As shown in Table 3, the odds of rural respondents reporting a behavior compared with urban respondents was not significantly different for any of the measures at any of the time points. However, as Table 4 shows, there were significant differences for some of the measures between time points. Among rural respondents, the odds of reporting adult visitors in the home in Fall 2020 or Winter 2021 were about half that in Spring 2020 (OR=0.50 and 0.53, respectively). Similarly the odds of visiting in the homes of others were lower in Winter 2021 compared to Spring 2020 for the respondent herself, her spouse, and her children (OR=0.33, 0.38, and 0.49); and in Winter 2021 compared to Fall 2020 for the respondent and her children (OR=0.42 and 0.54). In contrast, the odds of someone in the household attending church at Fall 2020 was 2.34 times the odds of church attendance in Spring 2020, and 2.86 for Winter 2021 compared to Spring 2020. Among urban residents, few differences between time points were significant. Households had 0.40 the odds of having adult visitors in Winter 2021 compared to Spring 2020. The odds ratio of church attendance in Fall 2020 compared to Spring 2020 was high (13.66), but the extreme odds ratio was likely due to only one respondent reporting church attendance in Spring 2020.

When attending large events was modeled, the rural by time interaction was significant, but had to be excluded from the model because odds ratios approached infinity. Therefore, it was only possible to estimate the odds of rural to urban for all three time points combined and for all respondents, both rural and urban, together across time points. For the former, across all three time points, the odds of a member of a rural household attending a social event was 1.91 times the odds (95% CI 0.89, 4.07) of a member of an urban household attending a social event. For the latter, the odds of someone in a household attending in Fall 2020 was 2.67 (95% CI 1.37, 5.22) times the odds of Spring 2020, but only 0.34 (95% CI 0.19, 0.63) times the odds in Winter 2021 compared to Fall 2020.

For the work measures, the rural by time interaction was significant, driven by the drastic decrease in risk at time 3 by the rural participants. The interaction term was kept in both models, allowing odds ratios to be calculated at different levels of time and rural/urban residence. The odds of a rural respondent being in the higher risk categories at work is higher than the odds of an urban participant being in the higher risk categories at work (Table 5). The odds ratio is significant in Fall 2020 (OR 2.41; 95% CI 1.09, 5.32), though not significant in Spring 2020 and Winter 2021. In Spring 2020, the odds of the rural participant’s spouse being in the higher risk work categories is 2.93 times the odds of an urban participant’s spouse being in the higher risk work categories. In Fall 2020, the odds ratio of being in the higher risk work categories for a rural participant’s spouse to an urban participant’s spouse becomes 2.47 (95% CI 0.99, 6.18). Among rural respondents, the odds of being in the higher risk work conditions in Fall 2020 is 1.75 the odds of being in the higher risk work conditions in Spring 2020 (95% CI 1.04. 2.95) (Table 6). However, the odds of a rural respondent being in the higher risk work conditions in Winter 2021 is 0.43 the odds of being in the higher risk work conditions in Fall 2020 (95% CI 0.24, 0.77). For spouses of rural respondents, the odds of being in the higher risk work conditions in Winter 2021 is 0.10 the odds of being in the higher risk category in Spring 2020 (95% CI 0.04, 0.25). Similarly, the odds of spouses being in the higher risk work conditions in Winter 2021 is 0.14 the odds of being in the higher risk conditions in Fall 2020 (95% CI 0.06, 0.32). Among urban respondents, none of the comparisons of work risk between time points are significant.

## 4. Discussion

In the absence of governmental protections, the COVID-19 pandemic continues to present essential workers and their families with the need to engage in practices to reduce exposure to the virus. Public health messaging in the US has been confusing, and the burden is often been put on individuals to evaluate and control their own risk [31]. Such a situation makes evaluating risk control behaviors over time in this marginalized population of interest.

As a group, these Latinx, primarily immigrant worker families largely reported specific personal practices indicating low or no risk for being exposed to COVID-19 at each of three time points in the year after the pandemic began. The exceptions to this are, for the most part, confined to possible exposures at work. When within-person practices were examined across the three time points and rural respondents were differentiated from urban, variability over time did emerge. Specifically, among rural residents, having visitors in the home or visiting in home of others declined over time, though attendance at church increased after Spring 2020. Attending large gatherings did not change over time for rural residents, but spiked up during Fall 2020 (which stretched into mid December) for urban residents.

In the workplace, Latinx rural mothers were more likely to be in a higher risk category than Latinx urban mothers, with the difference becoming significant in Fall 2020. Comparing time points, the increase in workplace risk for rural women in Fall compared to Spring 2020 is significant, with the risk falling to a level significantly lower in Winter 2021. For rural spouses, workplace risk was higher than for urban spouses in Spring and Fall 2020 than for Winter 2021. No seasonal differences were seen in urban spouses’ workplace risks.

These results do not seem to demonstrate a consistent time effect of individuals becoming more vigilant in their COVID-19 prevention practices over time, or becoming less vigilant, perhaps exhibiting “pandemic fatigue” over time. Rather, the findings seem to indicate structural forces related to work organization, economic pressures, and gender. These play out in the greater seasonality of work in rural compared urban communities, and gender differences in being in the labor force and in seasonality of work. Rural women in this study largely work as farm labor [21]. In the Spring of 2020, they experienced change in roles as children were out of school and child care centers were closed [28], leaving many with no choice but to stay home. They expressed concern, as their precarious economic status made work highly desirable, but impossible [28, 32]. By Fall 2020, children were back in school and women could respond to harvest labor demands. At the same time, spouses had ongoing work demands with Spring planting; these continued through Fall harvest. Regardless of any concerns about COVID-19 risk, this essential worker population generally experiences fear of losing their jobs if they cannot or do not work, as they are essential to their families’ economic survival [28, 33]. For both groups, Winter is traditionally a time of low employment in agriculture and economic difficulties and, to some extent, other outdoor industries such as construction.

The responses observed to these structural forces—to work even when protecting oneself is not possible—is supported by recent work on cultural supports for such behavior among the Latinx population in California [34]. Sobo and colleagues argue that the need to work and take care of family even while exposing oneself to risks of COVID-19 are rationalized by the concept of *aguarantismo*, which loosely translates as “to put up with” situations. This concept has been used in the context of dangerously deficient housing for Latinx farmworkers, as well [35].

These results support the notion that patterns of vigilance (and non-vigilance) surrounding COVID-19-related behaviors reflect structural forces rather than strictly individual choices [28, 36]. These structural forces include poverty, labor demand, and gendered work. These behaviors appear to have support of cultural values.

### 4.1. Limitations and Strengths

These findings should be interpreted in light of their limitations. The sample size is relatively small, and the sample was not selected specifically for a study of COVID-19 risk behaviors. It is limited to Latinx families living in North Carolina, so may not be generalizable to other locations. Data were collected using self-reports rather than observations.

Nevertheless, data were collected on this marginalized population during the early months of the COVID-19 pandemic. They represent essential work families, and, unlike most existing studies [e.g., 22, 26-27], represent repeated measures rather than cross-sectional design. Also unlike most existing studies [e.g., 25], this study obtained specific data on circumstances at home, in the community, and at work for the families represented, thus allowing a more complete picture of situational compliance with ways of reducing COVID-19 exposures. The study also included both rural and urban families. Most studies of COVID-19 risk behaviors in rural populations are from outside the US.

## 5. Conclusions

This study demonstrated that change over time in compliance with recommended practices to prevent COVID-19 reflects the living and working situations of essential Latinx families, rather than the gradual relaxation of vigilance that might be expected with long term health risks from the pandemic. The contrast between rural and urban worker families and men and women workers brings out the structural work-related issues faced by these families and workers. As with essential workers in general, not having options to quit work or work from home limits what these study participants could do as the pandemic progressed. These findings reinforce the belief widely held in occupational safety and health that protecting workers from hazards (such as exposure to COVID-19) must be top of mind for the industries and companies in which they work and not left to individual workers.

Further research is needed in the Latinx population on situational compliance with public health recommendations for COVID-19. In particular, research is needed in other parts of the US with different segments of the Latinx population.

## Figures and Tables

**Table 1.** Individual and household characteristics of participants in COVID-19 telephone surveys, Comparing Latinx rural and urban adults in North Carolina

	Rural (n=67)		Urban (n=44)		P value
	n	%	n	%	
Age at first available COVID-19 survey					0.1441
25 – 29 years	7	10.5	4	9.1	
30 – 34 years	24	35.8	8	18.2	
35 – 39 years	21	31.3	15	34.1	
40 – 47 years	15	22.4	17	38.6	
Country of birth (mother)					0.0146
Mexico	54	80.6	32	72.7	
El Salvador	7	10.4	0	0	
Guatemala	2	3.0	2	4.5	
Honduras	1	1.5	4	9.1	
Venezuela	0	0	2	4.5	
United States	3	4.5	4	9.1	
Language most comfortable for conversation					0.1515
Spanish	65	97.0	39	88.6	
English	1	1.5	4	9.1	
An indigenous language	1	1.5	1	2.3	
Highest level of education completed (mother)					0.2085
Less than sixth grade	13	19.4	4	9.1	
Sixth - eighth grade	18	26.9	9	20.5	
Ninth – eleventh grade	24	35.8	17	38.6	
High school or more	12	17.9	14	31.8	
Highest level of education completed (spouse)^1^					0.0349
Less than sixth grade	13	23.2	7	17.1	
Sixth - eighth grade	18	32.1	12	29.3	
Ninth – eleventh grade	20	35.7	9	21.9	
High school or more	5	8.9	13	31.7	

1 Totals 56 and 41, respectively, due to missing values.

**Table 2.** Comparison of number (%) of respondents reporting low, medium, or high risk behavior across three telephone surveys for Latinx rural and urban families.

	Rural Families			Urban Families		
	Spring 2020	Fall 2020	Winter 2021	Spring 2020	Fall 2020	Winter 2021
	n=67	n=63^1^	n=64	n=37	n=40	n=41
Adult visitors in home						
No risk	36 (53.7)	44 (69.8)	44 (68.8)	16 (43.2)	23 (57.5)	27 (65.9)
Any risk^2^	31 (46.3)	19 (30.2)	20 (31.2)	21 (56.8)	17 (42.5)	14 (34.1)
Child visitors in home						
No risk	39 (58.2)	42 (66.7)	45 (70.3)	23 (62.2)	30 (75.0)	25 (61.0)
Any risk	28 (41.8)	21 (33.3)	19 (29.7)	14 (37.8)	10 (25.0)	16 (39.0)
Self visit in other homes						
No risk	41 (61.2)	42 (66.7)	53 (82.8)	28 (75.7)	25 (62.5)	31 (75.6)
Any risk	26 (38.8)	21 (33.3)	11 (17.2)	9 (24.3)	15 (37.5)	10 (24.4)
Spouse visit in other homes						
No risk	33 (58.9)	39 (69.6)	45 (79.0)	26 (74.3)	28 (75.7)	35 (89.7)
Any risk	23 (41.1)	17 (30.4)	12 (21.0)	9 (25.7)	9 (24.3)	4 (10.3)
Children visit in other homes						
No risk	40 (59.7)	39 (61.9)	48 (75.0)	29 (78.4)	31 (77.5)	31 (75.6)
Any risk	27 (40.3)	24 (38.1)	16 (25.0)	8 (21.6)	9 (22.5)	10 (24.4)
Self worked with others						
Low risk^3^	37 (55.2)	25 (39.7)	40 (62.5)	22 (59.5)	25 (62.5)	27 (65.9)
Medium risk	29 (43.3)	38 (60.3)	23 (35.9)	12 (32.4)	15 (37.5)	14 (34.1)
High risk	1 (1.5)	0 (0.0)	1 (1.6)	3 (8.1)	0 (0.0)	0 (0.0)
Spouse worked with others^4^						
Low risk	3 (5.1)	2 (3.6)	14 (24.6)	4 (11.8)	3 (8.8)	6 (16.2)
Medium risk	35 (59.3)	39 (69.6)	41 (71.9)	24 (70.6)	27 (79.4)	28 (75.7)
High risk	21 (35.6)	15 (26.8)	2 (3.5)	6 (17.6)	4 (11.8)	3 (8.1)
Church attendance						
No risk	62 (92.5)	53 (84.1)	52 (81.3)	36 (97.3)	29 (72.5)	34 (82.9)
Any risk	5 (7.5)	10 (15.9)	12 (18.7)	1 (2.7)	11 (27.5)	7 (17.1)
Event attendance						
No risk	58 (86.6)	47 (74.6)	53 (82.8)	34 (91.9)	30 (75.0)	41 (100.0)
Any risk	9 (13.4)	16 (25.4)	11 (17.2)	3 (8.1)	10 (25.0)	0 (0.0)

1 N=63 instead of 64 due to missing data for one participant.

2 Includes medium and high risk.

3 Includes no risk and low risk.

4 Sample sizes lower for this variable because 7 participants did not know mask wearing in spouse’s workplace.

**Table 3.** Odds ratios (95% CI) of social interactions comparing rural to urban families, across three telephone surveys for Latinx families.

	Time 1: Spring 2020		Time 2: Fall 2020		Time 3: Winter 2021	
	Rural to Urban		Rural to Urban		Rural to Urban	
	OR (95% CI)	p-value	OR (95% CI)	p-value	OR (95% CI)	p-value
Adult visitors in home	0.66 (0.29, 1.47)	0.3069	0.58 (0.26, 1.33)	0.2023	0.88 (0.38, 2.02)	0.7571
Child visitors in home	1.18 (0.52, 2.69)	0.6942	1.50 (0.62, 3.64)	0.3702	0.66 (0.29, 1.51)	0.3233
Self visit in other homes	1.97 (0.80, 4.84)	0.1378	0.83 (0.36, 1.91)	0.6657	0.64 (0.25, 1.69)	0.3701
Spouse visit in other homes	2.01 (0.80, 5.08)	0.1386	1.36 (0.53, 3.48)	0.5265	2.33 (0.69, 7.86)	0.1716
Child visit in other homes	2.45 (0.97, 6.16)	0.0573	2.12 (0.86, 5.21)	0.1017	1.03 (0.42, 2.57)	0.9437
Church attendance	2.9 (0.33, 25.84)	0.3393	0.50 (0.19, 1.31)	0.1577	1.12 (0.40, 3.13)	0.8277

**Table 4.** Odds ratios (95% CI) of social interactions comparing by time points for rural to urban families, across three telephone surveys for Latinx families.

Rural						
	Fall to Spring 2020		Winter 2021 to Spring 2020		Winter 2021 to Fall 2020	
	OR (95% CI)	p-value	OR (95% CI)	p-value	OR (95% CI)	p-value
Adult visitors in home	0.50 (0.27, 0.93)	0.0295	0.53 (0.29, 0.98)	0.0416	1.05 (0.61, 1.81)	0.8533
Child visitors in home	0.70 (0.37, 1.32)	0.2676	0.59 (0.31, 1.10)	0.0987	0.84 (0.45, 1.58)	0.5954
Self visit in other homes	0.79 (0.45, 1.37)	0.4019	0.33 (0.18, 0.60)	0.0003	0.42 (0.22, 0.80)	0.0084
Spouse visit in other homes	0.63 (0.33, 1.17)	0.1424	0.38 (0.22, 0.68)	0.001	0.61 (0.32, 1.17)	0.1353
Child visit in other homes	0.91 (0.55, 1.51)	0.7207	0.49 (0.29, 0.85)	0.0116	0.54 (0.30, 0.97)	0.0405
Church attendance	2.34 (1.06, 5.15)	0.0348	2.86 (1.12, 7.28)	0.0274	1.22 (0.73, 2.05)	0.4432
Urban						
	Fall to Spring 2020		Winter 2021 to Spring 2020		Winter 2021 to Fall 2020	
	OR (95% CI)	p-value	OR (95% CI)	p-value	OR (95% CI)	p-value
Adult visitors in home	0.56 (0.27, 1.17)	0.1259	0.40 (0.17, 0.92)	0.0322	0.70 (0.32, 1.53)	0.3741
Child visitors in home	0.55 (0.19, 1.55)	0.2558	1.05 (0.43, 2.55)	0.9117	1.92 (0.80, 4.59)	0.1421
Self visit in other homes	1.87 (0.70, 4.95)	0.2099	1.00 (0.38, 2.64)	0.9942	0.54 (0.24, 1.23)	0.1413
Spouse visit in other homes	0.93 (0.34, 2.51)	0.8840	0.33 (0.09, 1.17)	0.0857	0.36 (0.10, 1.28)	0.1128
Child visit in other homes	1.05 (0.41, 2.70)	0.9154	1.17 (0.45, 3.01)	0.7461	1.11 (0.39, 3.13)	0.8418
Church attendance	13.66 (1.97, 94.84)	0.0082	7.41 (0.82, 66.94)	0.0744	0.54 (0.21, 1.42)	0.2136

**Table 5.** Odds ratios (95% CI) of social interactions in workplace settings comparing rural to urban families, across three telephone surveys for Latinx families.

	Spring 2020		Fall 2020		Winter 2021	
	Rural to Urban		Rural to Urban		Rural to Urban	
	OR (95% CI)	p-value	OR (95% CI)	p-value	OR (95% CI)	p-value
Self worked with others	1.05 (0.45, 2.45)	0.9042	2.41 (1.09, 5.32)	0.0297	1.18 (0.52, 2.67)	0.6873
Spouse worked with others	2.93 (1.01, 8.49)	0.0472	2.47 (0.99, 6.18)	0.0535	0.57 (0.23, 1.44)	0.2325

**Table 6.** Odds ratios (95% CI) of social interactions in workplace settings comparing by time points for rural to urban families, across three telephone surveys for Latinx families.

	Rural					
	Fall to Spring 2020		Winter 2021 to Spring 2020		Winter 2021 to Fall 2020	
	OR (95% CI)	p-value	OR (95% CI)	p-value	OR (95% CI)	p-value
Self worked with others	1.75 (1.04, 2.95)	0.0344	0.75 (0.43, 1.29)	0.2966	0.43 (0.24, 0.77)	0.0046
Spouse worked with others	0.72 (0.34, 1.52)	0.3934	0.10 (0.04, 0.25)	<.0001	0.14 (0.06, 0.32)	<.0001
	Urban					
	Fall to Spring 2020		Winter 2021 to Spring 2020		Winter 2021 to Fall 2020	
	OR (95% CI)	p-value	OR (95% CI)	p-value	OR (95% CI)	p-value
Self worked with others	0.77 (0.43, 1.38)	0.3749	0.66 (0.35, 1.27)	0.2186	0.87 (0.54, 1.39)	0.5553
Spouse worked with others	0.86 (0.32, 2.29)	0.7629	0.51 (0.18, 1.44)	0.2028	0.59 (0.32, 1.10)	0.0997
